# A Circulating MicroRNA Signature Capable of Assessing the Risk of Hepatocellular Carcinoma in Cirrhotic Patients

**DOI:** 10.1038/s41598-017-00631-9

**Published:** 2017-03-31

**Authors:** Ya-Hui Huang, Kung-Hao Liang, Rong-Nan Chien, Tsung-Hui Hu, Kwang-Huei Lin, Chao-Wei Hsu, Chih-Lang Lin, Tai-Long Pan, Po-Yuan Ke, Chau-Ting Yeh

**Affiliations:** 1 Liver Research Center, Chang Gung Memorial Hospital, Linkou, Taoyuan, Taiwan; 2 Liver Research Unit, Keelung Chang Gung Memorial Hospital, Keelung, Taiwan; 3 Division of Hepatogastroenterology, Department of Internal Medicine, Kaohsiung Chang Gung Memorial Hospital and Chang Gung University College of Medicine, Kaohsiung, Taiwan; 4 College of Medicine, Chang Gung University, Taoyuan, Taiwan; 5 School of Traditional Chinese Medicine, Chang Gung University, Taoyuan, Taiwan; 6 Research Center of Industry of Human Ecology, Chang Gung University of Science and Technology, Taoyuan, Taiwan; 7 Department of Biochemistry & Molecular Biology and Graduate Institute of Biomedical Sciences, College of Medicine, Chang Gung University, Taoyuan, Taiwan; 8 Molecular Medicine Research Center, Chang Gung University, Taoyuan, Taiwan

## Abstract

With the availability of potent antiviral therapies, complete suppression of hepatitis B virus (HBV) replication and total eradication of hepatitis C virus (HCV) can now be achieved. Despite these advances, hepatocellular carcinoma (HCC) still develops in a substantial proportion of cirrhotic patients, suggesting that host factors remain critical. Dysregulation of miRNAs is noted in many cancers, and circulating miRNAs can be readily assayed. In this study, we aimed to develop a circulating miRNA signature to assess the risk of HCC in cirrhotic patients. We first discovered that HBV- and HCV-related cirrhotic patients had distinguishable circulating miRNA profiles. A cohort of 330 cirrhotic patients was then compared against a cohort of 42 early HCC patients with complete remission. A score comprising 5 miRNAs and a binary etiology variable was established that was capable of differentiating between these two groups (AUC = 72.5%, *P* < 0.001). The 330 cirrhotic patients were further stratified into high- and low-risk groups, and all patients were longitudinally followed for 752 (11–891) days. Of them, 19 patients developed HCC. The high-risk group had significantly higher cumulative HCC incidence (*P* = 0.038). In summary, a circulating miRNA-based score was developed that is capable of assessing HCC risks in cirrhotic patients.

## Introduction

Liver cirrhosis is a major sequel of chronic hepatitis in patients suffering from a prolonged period of persistent necroinflammation. Once progressing to liver cirrhosis, patients are at high risk of liver function decompensation and hepatocellular carcinoma (HCC). Because hepatitis B virus (HBV) and hepatitis C virus (HCV) are the most common etiologies for chronic hepatitis, these two viruses are the most important causes of liver cirrhosis, functional decompensation and HCC. Several predictive models have been built to estimate the risk of HCC in patients with chronic HBV or HCV infection. When applying these scoring systems, two important aspects should be carefully evaluated. First, one must identify the clinical stage of hepatitis in which these models have been built. Second, one must distinguish whether the models were established before or after the era of effective antiviral therapy.

Several virological predictors are associated with HCC risk in chronic hepatitis B^1, 2^. Accordingly, risk scores were proposed by research groups from different Asian areas^3–6^. However, because the patients’ data were collected before antiviral treatment was available, the effect of antiviral treatment was not considered. Additionally, cirrhotic patients were either excluded in the baseline or included as a small proportion of the subjects. A subsequent study attempting to understand the accuracy of these scoring systems in patients receiving antiviral treatment discovered that the independent predictors for HCC include only older age, liver cirrhosis and virological relapse^7^. With the availability of potent antivirals, virological relapses can now be completely prevented. A recent retrospective analysis for antiviral-treated chronic hepatitis B patients revealed that the virological factors were no longer useful for HCC prediction and that only cirrhosis and therapeutic methods were independent predictors^8^.

In treatment-naïve, HCV-infected patients, the risk factors for HCC included liver cirrhosis, coinfection with HBV or HIV, HCV viremia and HCV genotypes^9–13^. In a study assessing HCC risk in interferon-treated, chronic hepatitis C patients, the following factors were included to formulate a scoring system: host factors, HCV genotype and sustained virological response^14^. With the development of new direct antiviral agents, complete virological response can now be achieved in >95% of patients, regardless of HCV genotype. Therefore, in the post-antiviral era, virological factors are less effective in prediction of HCC risk. The risk of HCC could be reduced to a very low level in patients without liver cirrhosis. However, recent studies indicated that a substantial proportion of cirrhotic HBV patients still developed HCC even under effective antiviral treatment^15, 16^. Thus, there is still an urgent need for HCC prediction among patients with liver cirrhosis.

Over the past decade, dysregulation of microRNAs (miRNAs) was noted in many cancers and could serve as diagnostic or prognostic biomarkers^17^. In 2008, Lawrie *et al*. discovered that dysregulation of miRNAs in serum was similar to that in tumor tissues^18^, suggesting that circulating miRNAs could be powerful biomarkers. The differential expression of miRNAs in various types of liver diseases, including HCC, has been explored^19, 20^, and dysregulation of several circulating miRNAs in HCC patients was reported in recent years^21–25^. As circulating miRNAs are easy to detect and analyze, it is possible to establish a signature profile for HCC risk prediction. In this study, we enrolled a cohort of HBV- and/or HCV-related liver cirrhosis patients who were regularly followed in liver clinics with sporadic HCC development during follow-up. Another cohort of early HCC patients under complete remission was included for comparative analysis. Given that miRNAs are more stable in plasma compared with serum^26^, 28 circulating miRNA candidates obtained from a literature search were quantitatively assayed using plasma samples. The aims of this study were (i) to understand whether differential miRNA profiles could be identified between cirrhotic patients with different hepatitis viral etiologies and (ii) to build a prediction model for HCC risk in cirrhotic patients with viral hepatitis.

## Results

### Determination of a panel of 28 candidate circulating miRNAs

Three criteria were established for determination of miRNA candidates: (1) the miRNA was reported with a similar role (oncogene or tumor suppressor) in HCC in at least two reports in the literature; (2) the miRNA could be clinically correlated with HCC patient survival; and (3) the miRNA was readily detectable using stem-loop RT-qPCR. After a thorough literature search, 18 miRNAs were identified to satisfy these criteria (miR-21, miR-221, let-7g, miR-122, miR-139-5p, miR-203, miR-18a, miR-338-3p, miR-125b, miR-126, miR-199b-5p, miR-222, miR-223, miR-25, miR-26a, miR-192, miR-27a, and miR-124). Furthermore, 10 additional miRNAs (miR-155, miR-15a, miR-15b, miR-29a, miR-30b, miR-30c, miR-381, miR-432, miR-486-3p, and miR-876-5p) that were identified as postoperative prognostic predictors for HCC recurrence in our previous study were also included^27^. Thus, a total of 28 miRNAs were analyzed.

### Cross-sectional analysis of associations between circulating miRNA levels and HCC

**Figure 1:**
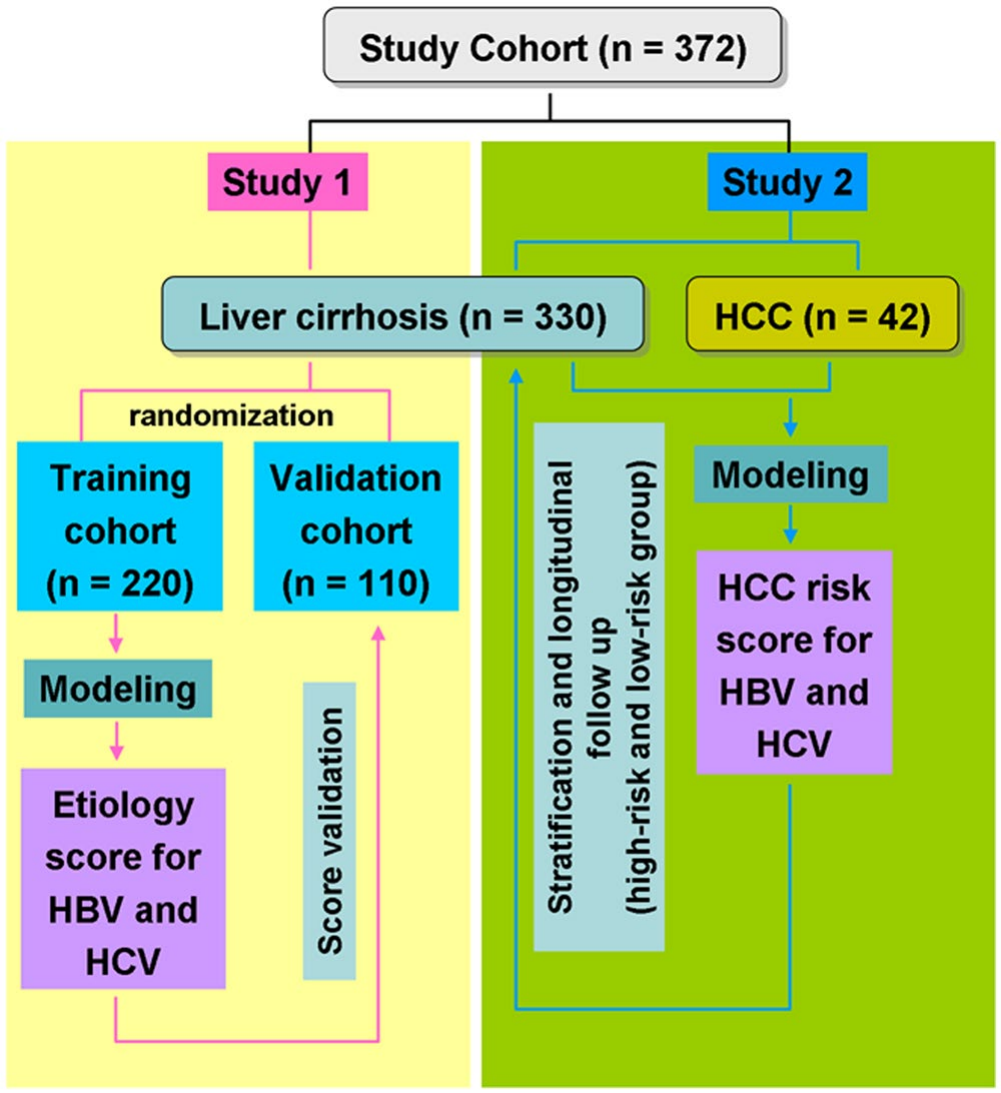
A flowchart of patient stratifications in this study.

The study cohorts comprised 330 liver cirrhotic patients without HCC at baseline and 42 patients with early HCC but under complete remission (Fig. 1). No significant disparity of gender, liver function variables (AST, ALT, bilirubin, AFP and albumin levels) and viral etiology (HBeAg positivity/negativity, Anti-HCV antibody positivity/negativity) was noted between the two groups (Table 1). In contrast, significant differences in miRNA levels between the cirrhosis and HCC groups were noted for 12 miRNAs, including miR-15a, miR-21, miR-486-3p, let-7g, miR-122, miR-18a, miR-338-3p, miR-222, miR-223, miR-26a, miR-192, and miR-124, using the univariate logistic regression method (Table 2). The multivariate analysis of these miRNAs revealed that only miR-15a was independently associated with HCC (Table 2). The adjusted odds ratio was 0.185 (95% confidence interval = 0.037~0.913), indicating that higher levels of miRNA were associated with reduced HCC risks.

**Table 1:** Demographic information of the study cohort.

Subject number	Cirrhosis	HCC	P
	330	42	0.001
Age, y	59.32 ± 10.86	64.6 ± 8.99	
Gender			
Male	226 (68.48%)	33 (78.57%)	0.181
Female	104 (31.52%)	9 (21.43%)	
Etiology			
HBsAg Positive	237 (71.82%)	30 (71.43%)	0.958
Anti-HCV Positive	110 (33.33%)	17 (40.48%)	0.358
Liver function variables			
AST, IU/L	43.02 ± 39.68	45.79 ± 36.14	0.647
ALT, IU/L	38.31 ± 45.02	37.26 ± 42.00	0.881
Bilirubin, mg/dL	1.40 ± 6.26	1.11 ± 0.67	0.437
AFP, ng/ml	10.48 ± 44.02	8.66 ± 11.80	0.559
Albumin, g/dL	3.60 ± 0.34	3.62 ± 0.41	0.733

**Table 2:** Association of miRNA levels with HCC using the logistic regression model.

	**Univariate**			**Multivariate**		
	Odds ratio	(95% CI)	P	Odds ratio	(95% CI)	P
miR-155	0.918	(0.541–1.559)	0.752			
miR-15a	0.474	(0.288–0.782)	**0.003**	0.185	(0.037–0.913)	**0.038**
miR-15b	0.752	(0.553–1.022)	0.069			
miR-21	0.633	(0.423–0.947)	**0.026**	2.891	(0.741–11.274)	0.126
miR-221	0.738	(0.494–1.103)	0.138			
miR-29a	0.751	(0.514–1.098)	0.140			
miR-30b	0.768	(0.551–1.071)	0.119			
miR-30c	0.767	(0.558–1.053)	0.101			
miR-381	1.061	(0.669–1.685)	0.800			
miR-432	0.960	(0.739–1.246)	0.758			
miR-486-3p	0.536	(0.301–0.953)	**0.034**	1.027	(0.419–2.518)	0.954
miR-876-5p	1.024	(0.632–1.661)	0.922			
let-7g	0.611	(0.407–0.917)	**0.017**	0.819	(0.274–2.445)	0.721
miR-122	0.525	(0.317–0.868)	**0.012**	0.662	(0.321–1.365)	0.264
miR-139-5p	0.686	(0.451–1.045)	0.079			
miR-203	0.957	(0.625–1.466)	0.840			
miR-18a	0.716	(0.526–0.974)	**0.033**	1.023	(0.523–2.001)	0.946
miR-338-3p	0.613	(0.387—0.972)	**0.038**	0.893	(0.445–1.790)	0.749
miR-125b	0.926	(0.531–1.615)	0.787			
miR-126	0.799	(0.627–1.018)	0.070			
miR-199b-5p	0.729	(0.442–1.204)	0.217			
miR-222	0.655	(0.455–0.943)	**0.023**	1.046	(0.269–4.074)	0.948
miR-223	0.754	(0.570–0.996)	**0.047**	0.943	(0.327–2.716)	0.913
miR-25	0.751	(0.458–1.231)	0.256			
miR-26a	0.682	(0.496–0.936)	**0.018**	0.894	(0.265–3.015)	0.857
miR-192	0.637	(0.412–0.986)	**0.043**	2.127	(0.760–5.947)	0.150
miR-27a	0.749	(0.555–1.011)	0.059			
miR-124	0.399	(0.204–0.782)	**0.007**	0.484	(0.199–1.173)	0.108

**Figure 2:**
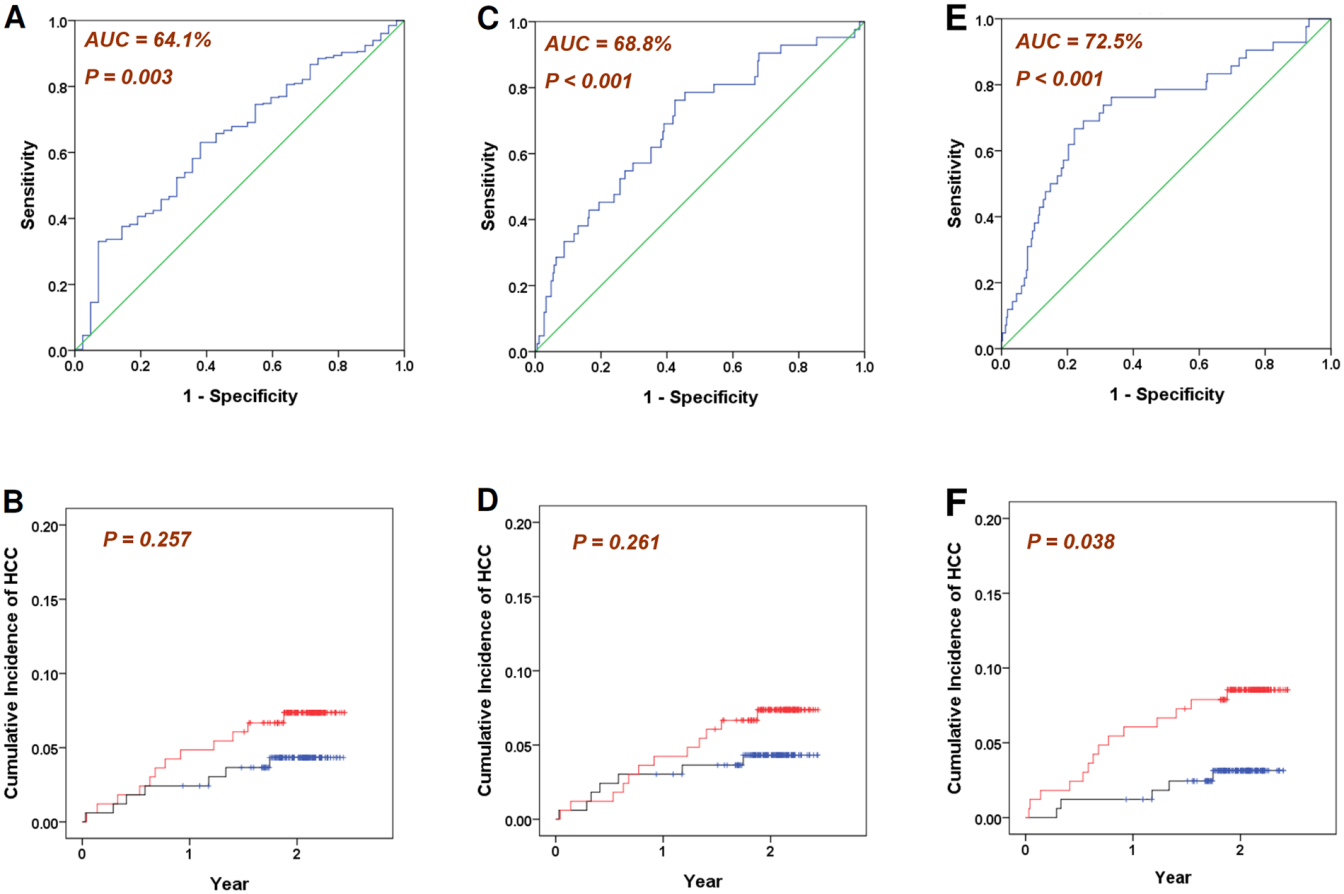
Cross-sectional classification and longitudinal time-to-HCC analysis of three different models. (**A**) and (**B**): based on miRNA-15a level only; (**C**) and (**D**): based on the logistic regression model incorporating 12 miRNAs; (**E**) and (**F**): based on the proposed HCC Risk score. Red: the cumulative incidence of the higher-risk patient stratum (N = 165); Blue: the cumulative incidence of the lower-risk patient stratum (N = 165).

The classification of cirrhosis and HCC patients based on their miR-15a levels was moderately successful, with an area under the receiver operating characteristic curve (AUC) of 64.1% (P = 0.003, Fig. 2A). However, when we analyzed the time-to-HCC development in cirrhotic patients after longitudinal follow-up (N = 330), the patient strata of high and low miR-15a levels (each strata N = 165) did not exhibit a significantly different cumulative incidence of HCC (P = 0.257, Fig. 2B).

We then incorporated all of the 12 miRNAs that exhibited significant differences in the univariate analysis into a logistic regression model for classifying cirrhotic and HCC patients. An AUC of 68.8% was achieved (P < 0.001, Fig. 2C). However, when the cirrhosis patients were stratified into the high-risk and low-risk groups by their estimated HCC risks in the logistic regression model (each group N = 165), no significant difference in the cumulative incidence of HCC was identified between the two groups (P = 0.261, Fig. 2D).

### Distinct circulating miRNA profiles between chronic hepatitis B and C in cirrhotic patients

In the cirrhotic group, 17 patients were co-infected by HBV and HCV; 220 patients had HBV monoinfection, and 93 had HCV monoinfection. We compared the miRNA expression profiles in three different classifications of etiology: (i) HBV monoinfection versus HCV monoinfection; (ii) HCV monoinfection versus HBV monoinfection + HBV/HCV co-infection; and (iii) HBV monoinfection versus HCV monoinfection + HBV/HCV co-infection. In (i), the levels of miR-15b (*P* = 0.022), miR-30b (*P* = 0.047) and miR-122 (*P* = 0.017) were significantly different (Table 3). In (ii), only miR-15b (*P* = 0.028) and miR-122 (*P* = 0.027) remained significantly different. In (iii), the number of differentially expressed miRNAs substantially increased to 14 (Table 2). The significance level of miR-15b (*P* = 0.020) and miR-122 (*P* = 0.009) also increased. As a result, classification method (iii) was used in the subsequent analysis.

**Table 3:** Different circulating miRNA levels were observed in liver cirrhotic patients with different viral etiology. Particularly, many miRNA have significant difference between “HBV monoinfection” and “HCV + co-infection” patients.

	HBV vs. HCV			HCV vs. HBV + co-infection			HBV vs. HCV + co-infection		
	AUROC	(95% CI)	P	AUROC	(95% CI)	P	AUROC	(95% CI)	P
miR-155	0.449	(0.377–0.521)	0.156	0.541	(0.469–0.612)	0.252	0.434	(0.367–0.501)	**0.049**
miR-15a	0.430	(0.360–0.500)	0.050	0.561	(0.492–0.631)	0.082	0.423	(0.357–0.489)	**0.023**
miR-15b	0.418	(0.348–0.488)	**0.022**	0.578	(0.508–0.648)	**0.028**	0.422	(0.356–0.487)	**0.020**
miR-21	0.435	(0.364–0.505)	0.068	0.555	(0.485–0.625)	0.121	0.421	(0.355–0.487)	**0.020**
miR-221	0.437	(0.368–0.506)	0.078	0.550	(0.482–0.619)	0.154	0.420	(0.355–0.484)	**0.017**
miR-29a	0.431	(0.362–0.499)	0.052	0.561	(0.493–0.629)	0.083	0.424	(0.359–0.488)	**0.024**
miR-30b	0.429	(0.360–0.498)	**0.047**	0.561	(0.493–0.629)	0.084	0.418	(0.353–0.483)	**0.015**
miR-30c	0.439	(0.369–0.508)	0.087	0.554	(0.486–0.623)	0.125	0.432	(0.366–0.498)	**0.044**
miR-381	0.536	(0.465–0.607)	0.311	0.456	(0.386–0.526)	0.212	0.512	(0.445–0.579)	0.721
miR-432	0.486	(0.415–0.557)	0.703	0.516	(0.445–0.586)	0.654	0.494	(0.427–0.560)	0.850
miR-486-3p	0.449	(0.378–0.519)	0.152	0.547	(0.478–0.617)	0.181	0.448	(0.382–0.514)	0.126
miR-876–5p	0.523	(0.453–0.593)	0.526	0.478	(0.408–0.548)	0.536	0.523	(0.457–0.589)	0.496
let-7g	0.440	(0.370–0.509)	0.092	0.549	(0.480–0.618)	0.168	0.424	(0.359–0.489)	**0.024**
miR-122	0.415	(0.345–0.485)	**0.017**	0.578	(0.509–0.648)	**0.027**	0.412	(0.347–0.477)	**0.009**
miR-139-5p	0.466	(0.397–0.535)	0.343	0.530	(0.461–0.599)	0.396	0.462	(0.397–0.528)	0.265
miR-203	0.454	(0.386–0.522)	0.201	0.546	(0.479–0.614)	0.193	0.460	(0.396–0.524)	0.240
miR-18a	0.438	(0.370–0.506)	0.083	0.555	(0.487–0.622)	0.122	0.432	(0.368–0.497)	**0.044**
miR-338-3p	0.460	(0.391–0.528)	0.258	0.538	(0.470–0.606)	0.282	0.460	(0.395–0.524)	0.232
miR-125b	0.485	(0.413–0.556)	0.665	0.503	(0.432–0.574)	0.938	0.456	(0.389–0.523)	0.192
miR-126	0.439	(0.371–0.507)	0.090	0.552	(0.484–0.619)	0.145	0.429	(0.365–0.493)	**0.035**
miR-199b-5p	0.473	(0.400–0.545)	0.449	0.517	(0.445–0.589)	0.635	0.454	(0.386–0.522)	0.171
miR-222	0.453	(0.385–0.522)	0.193	0.535	(0.467–0.603)	0.322	0.436	(0.371–0.500)	0.057
miR-223	0.446	(0.378–0.514)	0.131	0.543	(0.475–0.610)	0.227	0.431	(0.367–0.496)	**0.042**
miR-25	0.463	(0.392–0.533)	0.298	0.526	(0.456–0.595)	0.467	0.443	(0.377–0.510)	0.094
miR-26a	0.443	(0.375–0.512)	0.113	0.548	(0.480–0.616)	0.178	0.433	(0.368–0.498)	**0.048**
miR-192	0.452	(0.383–0.521)	0.181	0.537	(0.468–0.605)	0.299	0.437	(0.372–0.503)	0.064
miR-27a	0.474	(0.406–0.543)	0.475	0.515	(0.447–0.583)	0.675	0.458	(0.393–0.523)	0.215
miR-124	0.493	(0.422–0.565)	0.850	0.501	(0.431–0.572)	0.967	0.482	(0.415–0.549)	0.601

To formulate a miRNA profile to distinguish between HBV and HCV with/without HBV infection, the subjects were then randomly divided into training (n = 220; including 61 HCV, 146 HBV and 13 co-infected) and validation (n = 110; including 32 HCV, 74 HBV and 4 co-infected) subsets. No significant difference was observed between the miRNA levels in the two subsets (Table S1). In the training subset, a total of 7 miRNAs, including miR-21, miR-30c, let-7g, miR-15a, miR-122, miR-221 and miR-30b, reached *P* < 0.1 for the classification of etiology. Among them, 3 miRNAs had *P* < 0.05 (Table 4). The levels of seven miRNAs with *P* < 0.1 were then analyzed using the generalized iterative modeling (GIM) algorithm (see Methods) to formulate a model. Six out of the 7 miRNAs were chosen by the algorithm, and an etiology score was defined as follows:

1 $$\begin{array}{rcl}{\bf{Etiology}}\,{\bf{score}} & = & {\bf{miR}}-{\bf{21}}\cdot ({\bf{0}}{\rm{.}}{\bf{4724}})+{\bf{miR}}-{\bf{30c}}\cdot ({\bf{2}}{\rm{.}}{\bf{7896}})\\  &  & +\,{\bf{let}}-{\bf{7g}}\cdot ({\bf{0}}{\rm{.}}{\bf{6248}})+{\bf{miR}}-{\bf{15a}}\cdot {\bf{miR}}-{\bf{122}}\cdot (-{\bf{0}}{\rm{.}}{\bf{3175}})\\  &  & +\,{\bf{miR}}-{\bf{30b}}\cdot (-{\bf{3}}{\rm{.}}{\bf{1162}})+{\bf{1}}{\rm{.}}{\bf{6562}}\end{array}$$

**Table 4:** Univariate analysis of associations between miRNA levels and viral etiology. A total of 7 miRNAs reached a significance level of 0.1 (underscored). Among them, 3 miRNA has P < 0.05 (shown in bold face).

miRNA	**HBV vs. (HCV** **+** **co-infection)**			miRNA	**HBV vs. (HCV** **+** **co-infection)**		
	AUROC	(95% CI)	P		AUROC	(95% CI)	P
miR-155	0.459	(0.377–0.541)	0.320	miR-139-5p	0.457	(0.377–0.536)	0.293
miR-15a	0.421	(0.340–0.502)	0.055	miR-203	0.446	(0.368–0.524)	0.190
miR-15b	0.440	(0.358–0.521)	0.145	miR-18a	0.436	(0.357–0.515)	0.121
miR-21	0.414	(0.334–0.495)	**0.038**	miR-338-3p	0.487	(0.408–0.567)	0.759
miR-221	0.429	(0.350–0.508)	0.086	miR-125b	0.449	(0.369–0.529)	0.218
miR-29a	0.433	(0.353–0.513)	0.107	miR-126	0.441	(0.362–0.520)	0.156
miR-30b	0.419	(0.339–0.499)	0.050	miR-199b-5p	0.457	(0.375–0.539)	0.298
miR-30c	0.431	(0.352–0.511)	0.096	miR-222	0.436	(0.357–0.514)	0.119
miR-381	0.492	(0.411–0.572)	0.840	miR-223	0.437	(0.359–0.516)	0.129
miR-432	0.509	(0.428–0.590)	0.824	miR-25	0.438	(0.357–0.518)	0.130
miR-486-3p	0.436	(0.356–0.515)	0.120	miR-26a	0.451	(0.371–0.531)	0.237
miR-876-5p	0.517	(0.437–0.598)	0.673	miR-192	0.439	(0.359–0.519)	0.140
let-7g	0.418	(0.338–0.497)	**0.046**	miR-27a	0.452	(0.373–0.531)	0.241
miR-122	0.416	(0.337–0.494)	**0.041**	miR-124	0.477	(0.395–0.559)	0.578

In the training subset, the etiology model could classify patients with distinct etiology, achieving an AUC of 61.1% and a significance level of 0.007 (Supplementary Figure S1). The constant term of the model equation was calibrated so that the optimum cut-off, which was determined by Youden’s J statistic, occurred at a score of 0. Thus, a positive value of the etiology score indicated HCV-related cirrhosis (including coinfection), whereas a negative value indicated HBV-related cirrhosis. For prediction of HCV-related cirrhosis (including coinfection), the sensitivity was 67.57%, the specificity was 54.79%, the positive predictive value was 43.10%, and the negative predictive value was 76.92%.

**Figure 3:**
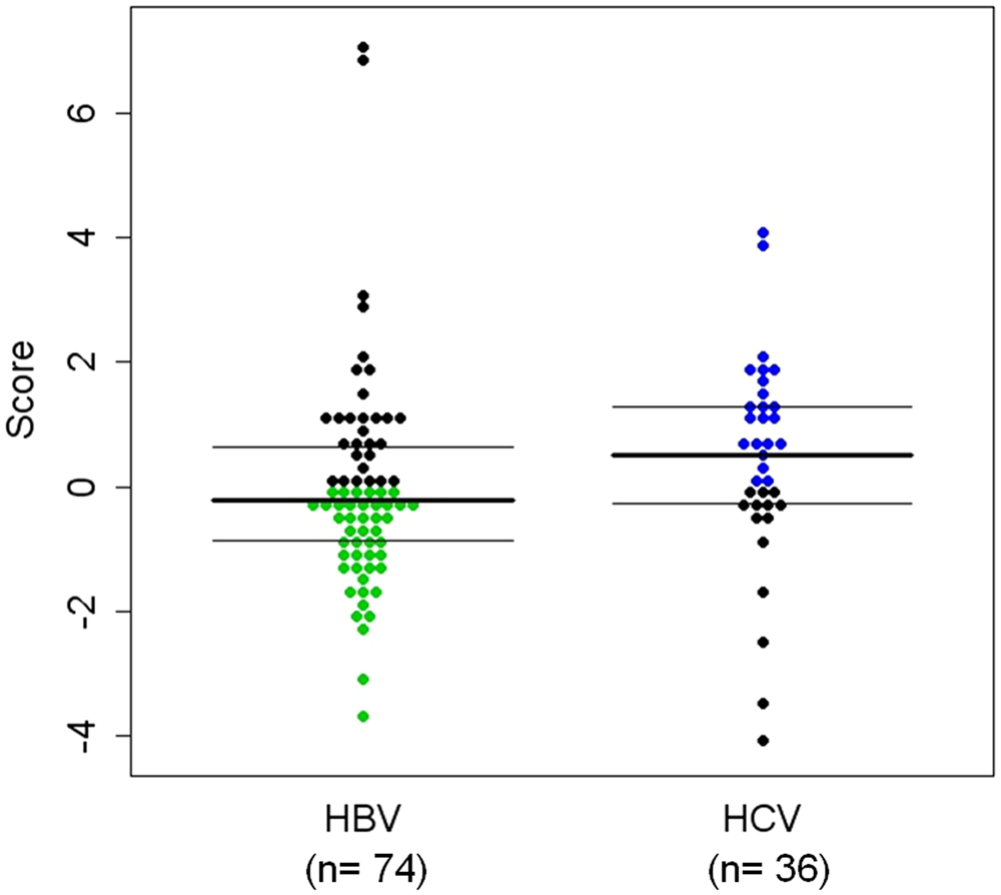
Distribution of the etiology-differentiation scores in the validation cohort. Each dot represents the score of a patient. Blue dots: HCV-related cirrhotic patients with score >0; Green dots: HBV-related cirrhotic patients with score ≤ 0. Median score for HBV-related cirrhotic patients = −0.230; Median score for HCV-related cirrhotic patients = 0.506; Mann-Whitney P = 0.017. When the cutoff was assigned as 0 for the prediction of HCV-related cirrhosis (including coinfection), the sensitivity was 61.11%, the specificity was 60.81%, the positive predictive value was 43.14%, and the negative predictive value was 76.27%; N = 110.

When the etiology model was tested in the validation subset, the score distributions of the HBV and HCV + co-infection groups remained significantly different (Mann-Whitney *P* = 0.017) (Fig. 3). The sensitivity was 61.11%, the specificity was 60.81%, the positive predictive value was 43.14%, and the negative predictive value was 76.27%.

### A miRNA signature for the prediction of subsequent HCC occurrence in cirrhotic patients who had no HCC at baseline

We then compared the miRNA levels in patients with (n = 42) and without (n = 330) HCC at baseline. A total of 16 miRNAs manifested significantly different levels in the univariate analysis, where the classification performance was assessed by the AUC and the Mann-Whitney U-statistics (Table 5). Based on the miRNA profiles differing significantly in chronic hepatitis B and C, a binary etiology variable “HCV positive” together with the 16 miRNAs was incorporated into the multivariate analysis by the GIM algorithm. If a patient was anti-HCV antibody positive, he/she was either HCV monoinfected or HCV-HBV co-infected, and the value of the variable was 1. In contrast, if a patient was anti-HCV antibody negative, the value of the variable was 0. An HCC risk score was then generated for the optimal distinction between HCC and non-HCC cirrhotic patients (AUC = 72.5%, *P* < 0.001, Fig. 2E).

2 $$\begin{array}{rcl}{\bf{HCC}}\,{\bf{Risk}}\,{\bf{Score}} & = & {\bf{HCV}}\,{\bf{positive}}\cdot {\bf{miR}}-{\bf{27a}}\cdot ({\bf{0}}{\rm{.}}{\bf{0135}}-{\bf{miR}}-{\bf{18a}}\cdot ({\bf{0}}{\rm{.}}{\bf{0029}}))\\  &  & -\,{\bf{miR}}-{\bf{18a}}\cdot ({\bf{0}}{\rm{.}}{\bf{0906}})-{\bf{miR}}-{\bf{222}}\cdot ({\bf{0}}{\rm{.}}{\bf{3534}})\\  &  & -\,{\bf{miR}}-{\bf{223}}\cdot ({\bf{0}}{\rm{.}}{\bf{007}})\\  &  & -\,{\bf{miR}}-{\bf{26a}}\cdot ({\bf{1}}{\rm{.}}{\bf{1716}}){\boldsymbol{+}}{\bf{miR}}-{\bf{27a}}\cdot ({\bf{1}}{\rm{.}}{\bf{4062}})\\  &  & {\boldsymbol{+}}\,{\bf{miR}}-{\bf{222}}\cdot {\bf{miR}}-{\bf{18a}}\cdot ({\bf{0}}{\rm{.}}{\bf{0150}})\\  &  & +\,{({\bf{miR}}-{\bf{222}})}^{{\bf{2}}}\cdot ({\bf{0}}{\rm{.}}{\bf{3842}}){\boldsymbol{+}}{({\bf{miR}}{\boldsymbol{-}}{\bf{26a}})}^{{\bf{3}}}\cdot ({\bf{0}}{\rm{.}}{\bf{0218}})\\  &  & -\,{({\bf{miR}}-{\bf{222}})}^{{\bf{2}}}\cdot {\bf{miR}}-{\bf{27a}}\cdot ({\bf{0}}.{\bf{0885}})\\  &  & +\,{({\bf{miR}}-{\bf{27a}})}^{{\bf{3}}}\cdot {({\bf{miR}}{\boldsymbol{-}}{\bf{18a}})}^{{\bf{2}}}\cdot ({\bf{0}}{\rm{.}}{\bf{0001}})-{\bf{1}}{\rm{.}}{\bf{1091}}\end{array}$$

where the “HCV positive” variable was defined as

$${\rm{HCV}}\,{\rm{positive}}=\{1,0|1:\mathrm{anti}-\mathrm{HCV}\,{\rm{antibody}}\,{\rm{positive}};0:\mathrm{anti}-\mathrm{HCV}\,{\rm{antibody}}\,{\rm{negative}}\}$$

**Table 5:** Univariate analysis of miRNA levels in association with HCC using the receiver operating characteristic curves. A total of 16 miRNAs has P < 0.05 (shown in bold face).

miRNA	Cirrhosis vs. HCC			miRNA	Cirrhosis vs. HCC		
	AUROC	(95% CI)	P		AUROC	(95% CI)	P
miR-155	0.465	(0.377–0.553)	0.464	miR-139-5p	0.435	(0.340–0.530)	0.170
miR-15a	0.359	(0.275–0.443)	**0.003**	miR-203	0.490	(0.399–0.580)	0.826
miR-15b	0.418	(0.332–0.504)	0.082	miR-18a	0.391	(0.304–0.477)	**0.021**
miR-21	0.394	(0.309–0.479)	**0.025**	miR-338-3p	0.376	(0.293–0.459)	**0.009**
miR-221	0.414	(0.326–0.502)	0.069	miR-125b	0.493	(0.406–0.580)	0.883
miR-29a	0.385	(0.307–0.462)	**0.015**	miR-126	0.384	(0.300–0.468)	**0.014**
miR-30b	0.408	(0.320–0.496)	0.052	miR-199b-5p	0.445	(0.351–0.539)	0.243
miR-30c	0.402	(0.315–0.489)	**0.038**	miR-222	0.377	(0.290–0.463)	**0.009**
miR-381	0.531	(0.447–0.614)	0.515	miR-223	0.382	(0.298–0.467)	**0.013**
miR-432	0.489	(0.384–0.595)	0.820	miR-25	0.420	(0.335–0.505)	0.091
miR-486-3p	0.394	(0.306–0.482)	**0.026**	miR-26a	0.389	(0.302–0.476)	**0.019**
miR-876-5p	0.503	(0.405–0.600)	0.956	miR-192	0.382	(0.299–0.465)	**0.013**
let-7g	0.386	(0.298–0.473)	**0.016**	miR-27a	0.403	(0.319–0.487)	**0.041**
miR-122	0.360	(0.270–0.449)	**0.003**	miR-124	0.359	(0.268–0.451)	**0.003**

By introducing the “HCV positive” variable, this model could be applied to both HBV- and HCV-related cirrhotic patients. Using the optimum cut-off determined by Youden’s J statistics to predict HCC occurrence, the sensitivity was 66.67%, the specificity was 77.88%, the positive predictive value was 27.72%, and the negative predictive value was 94.83%. The constant term of the model equation was calibrated to enable a zero median value in the cirrhosis patient group (N = 330).

Finally, because all 330 cirrhotic patients had been longitudinally followed, the initial HCC risk score could be calculated at the baseline when patients were enrolled. During the follow-up of 752 (11–891) days, 19 patients developed HCC. Comparing the distributions of HCC risk scores of those who did or did not develop HCC, a borderline significance level was obtained (P = 0.070, unpaired t-test with unequal variance). However, this analysis was a cross-sectional case/control analysis where the time information of HCC occurrence was not used. Therefore, we further analyzed the time-to-HCC development with respect to the patient strata by the baseline HCC risk scores. The patients were divided into the high-risk and low-risk groups (each N = 165). In total, 14 HCC development events were noted in the high-risk group, and 5 events were noted in the low-risk group. Our results indicated that the high-risk group exhibited significantly reduced time-to-HCC-development compared with the low-risk group (*P* = 0.038, Fig. 2F). The average times to HCC are 839 ± 14 days and 860 ± 8 days in the high-risk and low-risk groups, respectively.

As a benchmark, the same set of 16 miRNAs and the HCVpositive variable were jointly analyzed by the support vector machine (SVM) algorithm for the classification of the HCC and non-HCC groups. The resulting AUC was 68.3% (*P* < 0.001, Supplementary Figure S2A). In addition, the high-risk and low-risk patient strata by the SVM score (each strata N = 165) did not exhibit a significant difference in cumulative HCC incidence (*P* = 0.227, Supplementary Figure S2B). This benchmark showed that GIM HCC Risk Score outperformed the SVM score both in cross-sectional classification (AUC = 72.5% > 68.3%) and longitudinal analysis (P = 0.038 < 0.227).

### Improvement in the clinical-factor-based prediction model by incorporation of the miRNA score

Finally, we evaluated the well-established R.E.V.E.A.L. HCC model that was effective in predicting HCC risk in non-cirrhotic, treatment-naïve, chronic hepatitis B patients^3, 5^. We employed the same risk score assignment of three host variables: age (score increased 1 for every 5-year increment of age, starting from the minimum age of the cohort: 29), gender (male: score = 2, female: score = 0), and ALT level (≥45: score = 2; between 15 and 45: score = 1; <15: score = 0). The virological variables were not evaluated because our cirrhotic patients included both HBV- and HCV-infected patients. Additionally, all HBV patients received antiviral treatment, if needed, to suppress HBV-DNA to an undetectable level. This HCC risk score was a discrete score with integer values ranging from 0 to 14 (Supplementary Figure S3A). The median value was 8, which was used for patient stratification in the same manner as described in previous analyses. Two different cutoffs of patient stratification were evaluated. Comparing patients with score > 8 and ≤ 8, no significant difference in the cumulative incidence of HCC was found (log-rank P = 0.116, Supplementary Figure S3B). Alternatively, when patients were stratified using scores ≥ 8 and < 8, the high-risk and low-risk groups demonstrated different cumulative incidences of HCC (log-rank P = 0.018, Supplementary Figure S3C). Of the 191 patients identified as high risk, 16 (8.38%) developed HCC in 2 years.

To evaluate whether the simplified R.E.V.E.A.L. score and the miRNA HCC score were confounding variables with respect to HCC occurrence, we performed a multivariate logistic regression analysis on the two scores. Statistical significance was found in both the simplified R.E.V.E.A.L score (adjusted Wald-test P = 0.005) and the miRNA HCC score (adjusted P = 0.002), suggesting that they were independently associated with HCC occurrence (Supplementary Table S2).

The regression formula can also be used for the calculation of a combined score:

3 $$1.201\cdot ({\rm{miRNA}}\,{\rm{HCC}}\,{\rm{score}})+0.217\cdot ({\rm{simplified}}\,{\rm{REVEAL}}\,{\rm{score}})-3.892$$

The AUC of the combined score is 73.8%, a significant improvement from the simplified R.E.V.E.A.L. score (AUC = 66.4%, P = 0.034, Supplementary Figure S4A). In contrast, no significant difference was observed between the combined score and the miRNA score (AUC = 72.5%, P = 0.657, Supplementary Figure S4B). When the combined score was used for patient stratification, a significant difference in the cumulative incidence of HCC was observed between the high- and low-risk groups (P = 0.001, Supplementary Figure S4C).

We also explored the stratifications of patients using both the miRNA model and the simplified R.E.V.E.A.L. model (scores ≥ 8 versus < 8). Four distinct curves of the cumulative incidence of HCC were observed (log-rank P = 0.011, Supplementary Figure S5A). Furthermore, patients identified as the highest risk (in the high-risk group of both models; N = 98, 29.7% of all patients) manifested a distinct incidence curve from the other three groups (N = 232) (log-rank P = 0.001, Supplementary Figure S5B). Of these 98 patients, 12 (12.24%) developed HCC in 2 years. The combined model outperformed the simplified R.E.V.E.A.L. model as well as the miRNA-only model, which identified 165 patients as high risk, and 14 (8.48%) patients subsequently developed HCC.

## Discussion

Few predictive models for HCC occurrence are available for HBV- or HCV-related cirrhotic patients^3, 14, 28^. These models, however, included patients who had not received antiviral treatment (for HBV) or patients who had received interferon-based treatment (for HCV). Under these circumstances, virological factors are the key predictors. However, when the HBV models were validated in entecavir-treated patients, the only independent virological predictor was virological relapse^7^. As tenofovir was available globally, virological relapse could now be prevented in almost all HBV patients. However, with more effective direct antiviral agents for HCV, almost all HCV patients could be virologically cured. Thus, virological factors might not be included in the future prediction models in antiviral-treated patients. Despite effective antiviral treatment, HCC still developed in a substantial proportion of cirrhotic patients. It is therefore critical to develop HCC prediction models for this group of patients with or without antiviral treatments.

Instead of including virological factors, miRNAs were selected as candidate predictors in the study. Numerous miRNAs were aberrantly expressed in HCC^29^, and circulating miRNAs are readily detectable in serum or plasma^30^ at quantifiable levels by qPCR^31^. Given that HBV and HCV were the two most important etiologies for cirrhosis in our population, we first studied whether the circulating miRNA profiles were different between these two etiologies. The results showed that HBV- and HCV-related cirrhosis could be distinguished by specific miRNA profiling, suggesting that during the long courses of chronic hepatitis, HBV and HCV evoked different sets of miRNAs in the liver, both resulting in liver cirrhosis. Accordingly, a logistic variable was included in the prediction model for HCC risk assessment; therefore, this model could be used for both HBV- and HCV-related cirrhotic patients.

In this study, the model was built on 330 cirrhotic patients with no HCC developed at baseline. During the subsequent follow-up, some of these patients developed HCCs. To build a more accurate model, these HCC patients should be incorporated into the HCC group (n = 42). However, in the present study, we intended to perform a validation test using this cohort of patients for the established equation. Thus, these would-be HCC patients were included in the 330-patient cohort. Although the validation was successful, this was not a truly prospective study. An authentic prospective validation study should be conducted for a final conclusion.

To our knowledge, this is the first circulating miRNA-based model for HCC risk prediction in cirrhosis patients. Our studies provided supporting evidence for two interesting concepts. First, HBV and HCV evoked differential miRNA dysregulation during the long courses of chronic hepatitis toward liver cirrhosis. Second, dysregulation of miRNAs may have occurred prior to the development of HCC, and the baseline miRNA levels might be used for identifying high HCC-risk patients among liver cirrhotic patients. Combined with the conventional clinical predictors, including age, gender and baseline ALT levels, a subgroup of cirrhotic patients (~30%) was identified with a particularly high risk of HCC compared with other cirrhotic patients (P = 0.001).

## Materials and Methods

### Patients

This study was approved by the Institutional Review Board of Chang Gung Memorial Hospital, Taiwan (IRB No. 103-5039 C). Written informed consent was obtained from all patients, and the study was conducted in accordance with the Guidelines for Good Clinical Practice and the applicable laws and regulations. A total of 372 HBV- and/or HCV-related cirrhotic patients from three branches (Keelung, Linkou, and Kaohsiung Branches; located at the northern, central-northern, and southern parts of Taiwan, respectively) of Chang Gung Memorial Hospital were enrolled. All of the patients provided informed consent. Among them, 330 patients had liver cirrhosis but did not develop HCC at the time when patients were recruited (the liver cirrhosis group), whereas 42 patients were diagnosed as early HCC at the Barcelona Clinic of the Liver Cancer Stage A (the HCC group). These HCC patients were treated by either surgical removal or radiofrequency ablation and were under complete remission when recruited. Plasma samples were collected from these subjects for analysis of 28 circulating miRNAs, which were obtained from a literature search. The liver cirrhosis group was further divided into the training and validation subsets by a 2:1 randomization for evaluation of miRNA profiles capable of distinguishing viral etiologies (Fig. 1). All 330 cirrhotic patients were prospectively followed until development of HCC or the final date of follow-up on 2015/07/23, whichever came first. Patients who did not develop HCC by the end of the follow-up were considered right-censored data in the time-to-HCC analysis. The median follow-up period was 752 (11–891) days.

HCC was diagnosed by cytology or liver biopsy. Liver cirrhosis was diagnosed by either liver biopsy or ultrasound characteristics (coarse parenchyma and uneven surface) plus at least one of the following: (i) endoscopy visualization of esophageal varices, (ii) fibroscan value > 12kPa, or (iii) aspartate transaminase (AST) to platelet ratio index > 1.

No HCV-related cirrhotic patient received antiviral treatment at the time of, or after, enrollment. All HBV-related cirrhotic patients had a serum HBV-DNA level < 500 IU/mL. In 34 patients who had HBV-DNA level > 2000 IU/mL before enrollment, life-long antiviral treatment was provided, so that when included, the HBV-DNA levels were < 500 IU/mL.

### RNA Extraction

To avoid miRNA degradation, 250 μL of plasma sample was mixed with 750 μL of TRIzol LS reagent (Thermo Fisher Scientific, Wilmington, DE, USA) immediately after centrifuge separation from blood cells. The RNA-containing mixture was transferred to a prepared PLG Heavy tube (BIOTOOLS, New Taipei City, Taiwan) for RNA extraction following the procedure provided by the manufacturer.

### MicroRNA Detection

A stem-loop RT-qPCR method was performed as described in our previous report^27^. Briefly, 10 μl RT reaction mixture containing miRNA-specific stem-loop RT primers (final concentration, 2 nM each), 500 μM dNTP, 0.5 μl MMLV HP RT EPICENTRE Biotechnologies, Madison, WI), 0.5 μl RNaseOut (Invitrogen), and 80 ng total RNA was used for the RT reaction performed at 16 °C for 30 min, followed by 50 cycles of reaction at 20 °C for 30 s, 42 °C for 30 s, and 50 °C for 1 s. The RT products were diluted 8-fold before qPCR. Next, 0.5 μl of diluted RT product was used as a template in a 6-μl PCR reaction mixture that contained 1× SYBR Master Mix (Applied Biosystem, Foster City, CA), 200 nM miRNA-specific forward primer, and 200 nM universal reverse primers. The conditions for qPCR were 95 °C for 10 min, followed by 40 cycles of reaction at 95 °C for 15 s and 63 °C for 32 s. ABI 7900HT Fast Real-Time PCR system (Foster City, CA) was used for qPCR reactions. ABI 7900HT SDS 2.3 software was used to calculate the threshold cycle (Ct) and relative quantification. The ΔCt method was used to calculate expression levels normalized against U6. The miRNA expression level was calculated as POWER(2, ΔCt) × 10^6^.

### Statistical Analysis

Cross-sectional clinical variables, such as etiologies or logarithmic transformed miRNA levels, were evaluated using the area under the receiver operating characteristic curves (AUC), which were estimated by non-parametric empirical calculations using the SPSS statistical software version 21 (IBM, New York City, NY). The significance levels were evaluated using Mann-Whitney U statistics^32^. Longitudinal analysis of time to HCC was performed by the Kaplan-Meier method. Statistical significance was evaluated using the non-parametric log-rank test. Classification by the support vector machine was performed with the radial basis function kernel, using the svm() function of the R statistical scripting language. Differences of AUCs of two correlated, empirical ROC curves were evaluated by a bootstrap test with 2000 times of re-sampling, using the pROC package^32^ of the R runtime environment.

### Generalized Iterative Modeling

A multivariate modeling method, the generalized iterative modeling (GIM) method, was used to produce an algebraic biosignature model (*M*) for the optimum clinical classification in terms of the maximum AUC:

4 $$\forall \,M:arg{\max }_{M}{\rm{AUC}}(M),$$

where the AUC of *M* equates to the non-parametric Mann-Whitney U-statistics, normalized by the numbers of patients in two distinct clinical classes, *n1* and *n2* ^33^:

5 $$\mathrm{AUC}\,(M)=\frac{Mann-Whitney\,U\,statistics}{n1\cdot n2}$$

GIM is a generalization of previously published algorithms, GABA and HABA, which were specifically designed for analyzing discrete genomic information and therefore were restricted to Boolean algebra^34, 35^. The generalized algorithm can currently incorporate both continuous clinical variables and discrete genomic variables altogether. Briefly, candidate biosignature models were produced by joining randomly-selected clinical parameters with three basic algebraic operations, addition (+), subtraction (−) and multiplication (·). These models were then sculptured progressively to generate new models by the following computational operations: coefficient adjustment, adding or removing clinical variables, changing the algebraic operators between variables, and a crossover of two candidate models. Each model was assessed by their classification performance gauged by the empirical AUC. Models with better performance were more likely to be retained in the subsequent computation. The entire process was iterated until quasi-optimal models were identified when the AUC did not increase any further after a predefined number of iterations. An optional input variable of the algorithm, the cost of model complexity *c* (c ≥ 0), was also introduced to penalize candidate models with many variables. In the computation of the HCC_Risk_Score, the value of *c* is 0.0005. The pseudocode of the GIM algorithm is as follows:

**Input**

*T*: a patient-by-variable matrix

*L*: a vector of patients’ class labels

*c*: the cost of model complexity, c ≥ 0.

**Output**

 A model *M1*

**Procedure GIM()**

generate a set of *k* models (*S*) randomly;

*S* = {*Mi* | *M1*, *M2*, *M3*……*Mk*}

**for** each *Mi* **in** *S*

{

**for** each patient in *T*

  calculate the score by *Mi*

 calculate *AUC* based on the scores of all patients in *T*, and the labels *L*

 Fitness score *F*(*Mi*) = *AUC* (*Mi*) *−* c * (number of variables in *Mi*)

}

**sort** the models in *S w.r.t*. descending *F*(*Mi*)

**Repeat**

**{**

**pick** the subset *Sp* ∈ *S* with higher *F*

*Sd* = *S* − *Sp*

**discard** *Sd*

 Generate *Sd’* by the following methods

 {

  Randomly pick *M* ∈ *Sp* and perform one of the following 

  {

  Coefficient adjustment 

  Adding/removing variables 

  Changing the algebraic operators between variables

   from multiplication (·) to addition (+), or  

   from addition (+) to multiplication (·) 

  } 

  Randomly pick *Mi, Mj* ∈ *Sp* and perform  

   Crossover between *Mi* and *Mj*

**}**

*Update S* = *Sp* + *Sd*’

**for** each *Mi* in *S*

 { 

**for** each patient in *T*

   calculate the score by *Mi*

  calculate *AUC* based on the scores of all patients in *T*, and the labels *L*

   Fitness score *F*(*Mi*) = *AUC* (*Mi*) − c * (number of variables in *Mi*)

 }

**sort** the models in *S w.r.t*. descending *F*(*Mi*)

**} until** (*AUC* (*M1*) =  = 1 or timeout)

**return** (*M1*);

## Electronic supplementary material

Supplementary Info (clean version)
