# Supplementary material for: A Circulating MicroRNA Signature Capable of Assessing the Risk of Hepatocellular Carcinoma in Cirrhotic Patients
Source: Sci Rep. 2017 Mar 31;7:523. doi: 10.1038/s41598-017-00631-9 (PMC5428873; doi:10.1038/s41598-017-00631-9)
Supplement: Supplementary file 1 — Supplementary Info (clean version) [file 41598_2017_631_MOESM1_ESM.pdf]

Supplementary Information

A Circulating MicroRNA Signature Capable of Assessing the Risk of Hepatocellular Carcinoma in Cirrhotic Patients

Ya-Hui Huang[1,+], Kung-Hao Liang[1,+], Rong-Nan Chien[2], Tsung-Hui Hu[3], Kwang-Huei Lin[1,4], Chao-Wei Hsu[1,4], Chih-Lang Lin[1,2], Tai-Long Pan[5,6], Po-Yuan Ke[1,7] and Chau-Ting Yeh[1,4,8,*]

[1] Liver Research Center, Chang Gung Memorial Hospital, Linkou, Taoyuan, Taiwan

[2] Liver Research Unit, Keelung Chang Gung Memorial Hospital, Keelung, Taiwan

[3] Division of Hepatogastroenterology, Department of Internal Medicine, Kaohsiu Chang Gung Memorial Hospital and Chang Gung University College of Medicine, Kao-hsiung, Taiwan

[4] College of Medicine, Chang-Gung University, Taoyuan, Taiwan

[5] School of Traditional Chinese Medicine, Chang-Gung University, Taoyuan, Taiwan

[6] Research Center of Industry of Human Ecology, Chang-Gung University of Science and Technology, Taoyuan, Taiwan

[7] Department of Biochemistry & Molecular Biology and Graduate Institute of Biomedical Sciences, College of Medicine, Chang Gung University, Taoyuan, Taiwan

[8] Molecular Medicine Research Center, Chang-Gung University, Taoyuan, Taiwan

[+]These authors contribute equally to this work.

Table S1. miRNA levels of the training (n = 220) and validation (n = 110) groups. 2:1 randomization were performed to divide the dataset into two partitions. No significant difference was found between the two partitions.

	Training		Validation		*P*
	Median	(min , max)	Median	(min , max)	
miR-155	3.629	(2.114 , 6.263)	3.694	(1.805 , 5.280)	0.284
miR-15a	4.326	(2.414 , 5.647)	4.411	(2.519 , 6.026)	0.503
miR-15b	5.060	(1.692 , 6.722)	4.890	(1.385 , 6.262)	0.606
miR-21	5.064	(1.196 , 6.456)	5.101	(2.561 , 6.799)	0.638
miR-221	5.549	(3.173 , 7.031)	5.527	(3.317 , 7.319)	0.867
miR-29a	4.234	(1.458 , 5.647)	4.255	(1.789 , 5.685)	0.738
miR-30b	5.281	(2.545 , 7.508)	5.342	(2.766 , 7.207)	0.933
miR-30c	5.363	(2.646 , 7.121)	5.456	(1.883 , 7.699)	0.729
miR-381	4.124	(2.088 , 6.389)	4.103	(2.309 , 6.500)	0.454
miR-432	3.996	(0.538 , 7.282)	3.875	(1.467 , 6.337)	0.259
miR-486-3p	3.981	(2.588 , 5.188)	3.996	(1.932 , 5.906)	0.648
miR-876-5p	3.747	(2.124 , 6.717)	3.794	(1.956 , 5.829)	0.970
let-7g	4.995	(3.066 , 6.565)	5.052	(3.117 , 7.029)	0.980
miR-122	4.026	(2.328 , 6.132)	4.057	(2.048 , 5.933)	0.981
miR-139-5p	4.648	(2.399 , 6.011)	4.611	(2.510 , 6.568)	0.713
miR-203	2.490	(0.356 , 5.183)	2.415	(0.486 , 4.455)	0.239
miR-18a	4.003	(1.010 , 6.111)	4.060	(0.486 , 5.486)	0.980
miR-338-3p	2.555	(0.494 , 6.130)	2.555	(0.486 , 4.191)	0.810
miR-125b	4.489	(2.848 , 6.802)	4.506	(3.039 , 6.005)	0.292
miR-126	5.556	(1.344 , 7.293)	5.631	(1.385 , 7.612)	0.995
miR-199b-5p	3.189	(1.248 , 6.963)	3.232	(1.735 , 4.898)	0.386
miR-222	4.977	(2.001 , 6.489)	4.974	(2.636 , 6.863)	0.954
miR-223	6.554	(3.038 , 8.139)	6.567	(3.507 , 8.220)	0.902
miR-25	5.823	(3.630 , 7.276)	5.899	(3.875 , 7.889)	0.226
miR-26a	5.307	(2.273 , 7.046)	5.380	(2.838 , 7.241)	0.983
miR-192	3.648	(1.125 , 5.178)	3.694	(1.289 , 5.484)	0.393
miR-27a	4.962	(1.123 , 7.072)	4.942	(2.201 , 7.069)	0.718
miR-124	3.844	(1.423 , 5.109)	3.858	(2.744 , 5.510)	0.390

Table S2. Multivariate logistic regression analysis of the miRNA-HCC score and the simplified R.E.V.E.A.L. HCC score for the classification of cirrhotic and HCC patients.

	Multivariate logistic regression			
	Beta	Odds ratio	(95% CI)	P
miRNA-HCC score	1.201	3.323	(1.564 — 7.063)	**0.002**
REVEAL score	0.217	1.242	(1.068 — 1.445)	**0.005**

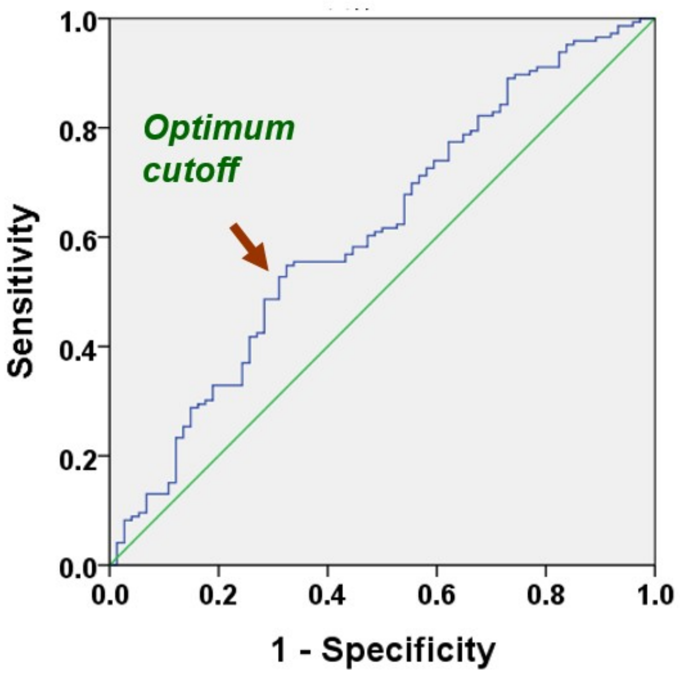

Figure S1. The receiver operating characteristic curve of the etiology score in the classification of cirrhotic patients with distinct etiologies in the training dataset (N = 220). The area under the curve is 61.1%, P = 0.007. The brown arrow indicated the optimum cutting point when the score is 0, where the Youden's J statistics is maximized.

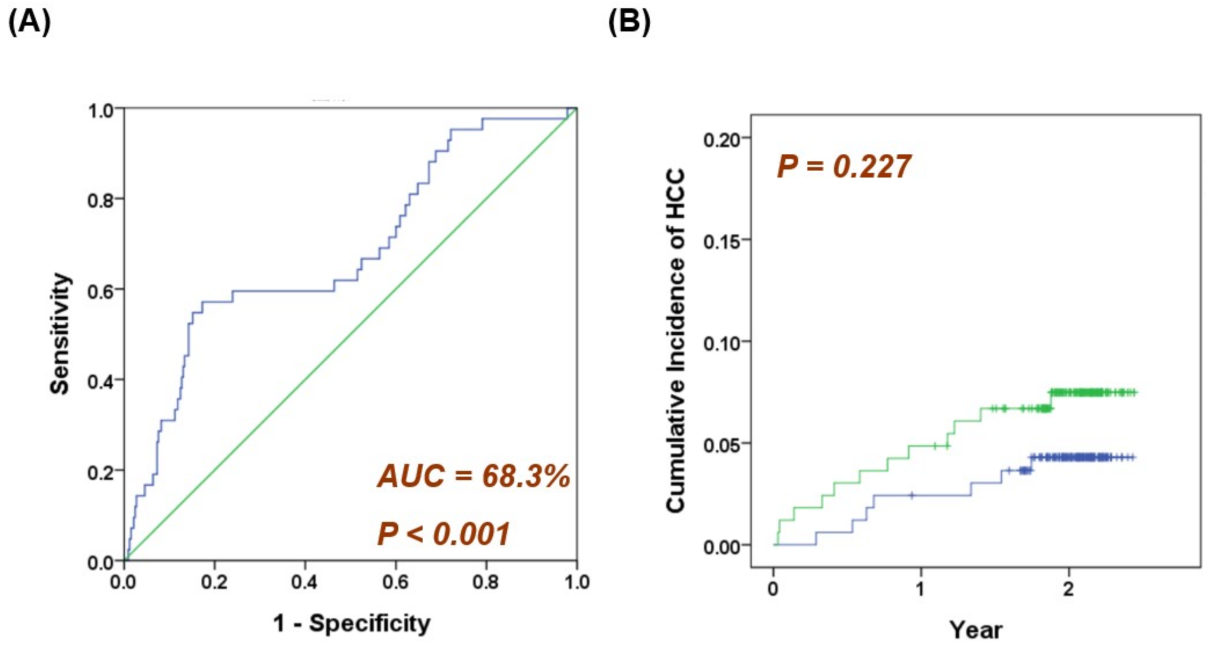

Figure S2. Cross-sectional classification and longitudinal time-to-HCC analysis of the support vector machine model. (A) Cross-sectional classification; (B) Longitudinal time-to-HCC analysis. Green: the cumulative HCC incidence of the higher-risk patient stratum (N = 165); Blue: the cumulative HCC incidence of the lower-risk patient stratum (N = 165).

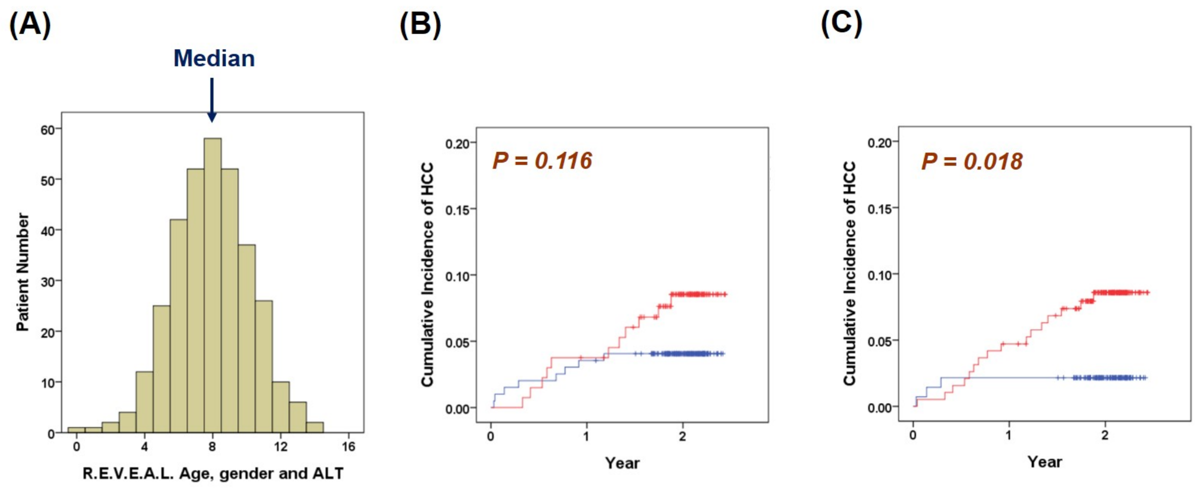

Figure S3. The evaluation of HCC risks using the age, gender and ALT elements in the R.E.V.E.A.L. model. (A) The risk score distribution of the cirrhosis patient cohort (N = 330). The median value was 8. (B) The Kaplan-Meier plot of the cumulative incidence of HCC in the high- and low-risk groups shown by red and blue colors respectively. The high-risk group was defined when score > 8 (N = 133), while the low-risk group was defined as score ≤ 8 (N= 197). No statistically significant difference in the cumulative incidence of HCC was found between the two groups (log-rank P = 0.116). (C) The high-risk group was defined when score ≥ 8 (N = 191), while the low-risk group was defined as score < 8 (N= 139). Significant difference was found between the two groups (log-rank P = 0.018).

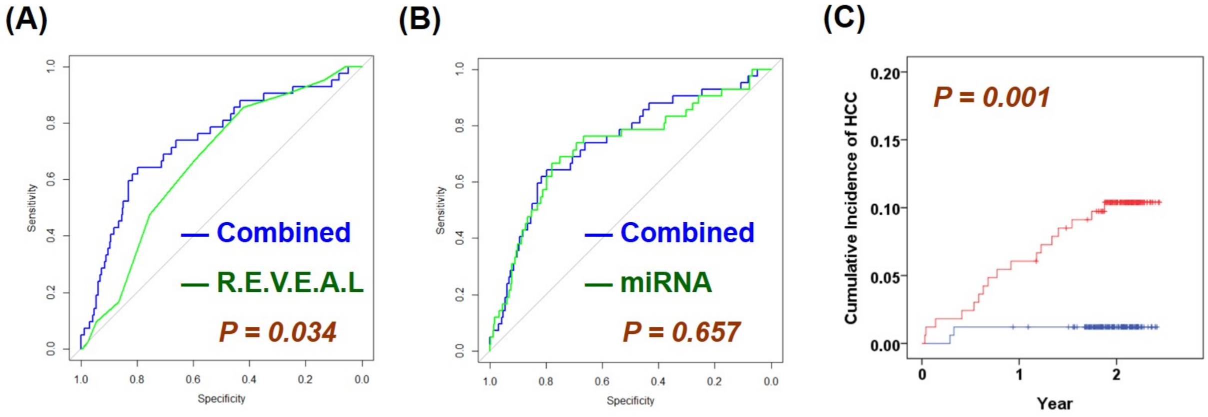

Fig S4. Evaluation of the combination of simplified R.E.V.E.A.L. score and the miRNA HCC score using the multivariate logistic regression method. (A) The ROC curves of the simplified R.E.V.E.A.L. score (AUC = 66.4%) and the combined score (AUC = 73.8%) for the classification of liver cirrhotic and HCC patients. Significance level of the differences between the two ROC curves was 0.034, estimated by a bootstrap test with 2000 times of re-sampling. (B) No significant difference was found between the ROC curves of the miRNA score (AUC = 72.5%) and the combined score (AUC = 73.8%, P = 0.657). (C) The Kaplan-Meier plot of the cumulative incidence of HCC of high- and low-risk patients (N = 165 for each group) based on the combined score. Significant difference was found between the two groups (log-rank P = 0.001).

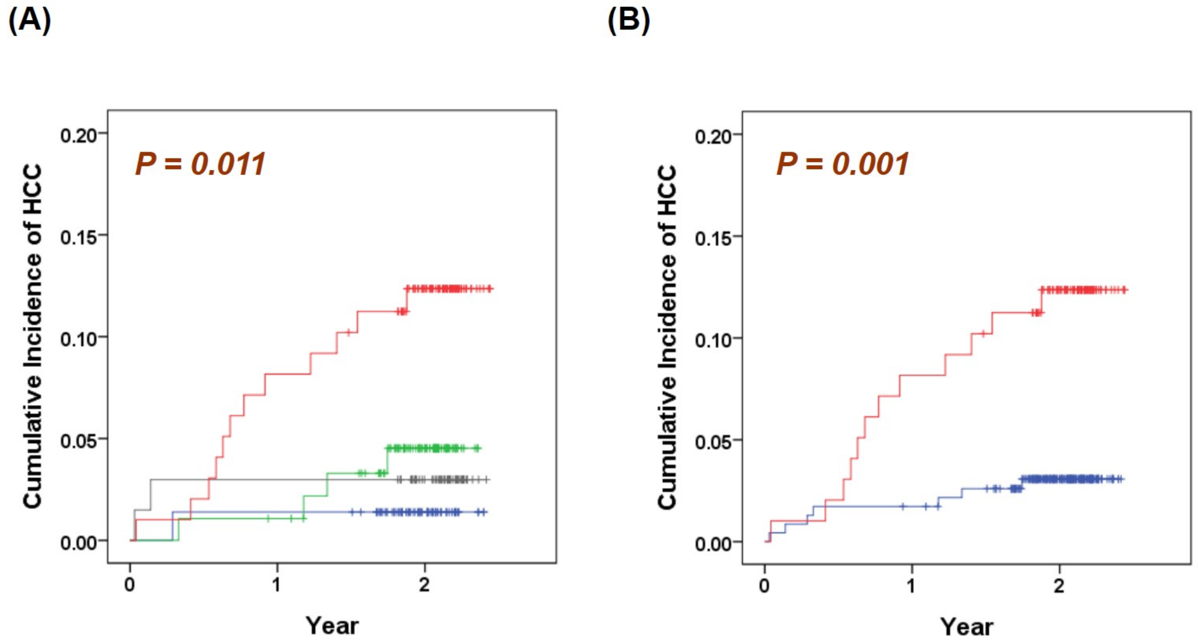

Figure S5. The Kaplan-Meier plots of patient strata by use of both the miRNA model and the R.E.V.E.A.L-age-gender-ALT model (score ≥ 8 vs. < 8). (A) Red: patients identified as high-risk in both models (N = 98). Gray: patients identified as high-risk in the miRNA model but as low-risk in the R.E.V.E.A.L. model (N = 67); Green: patients identified as low-risk in the miRNA model but as high-risk in the R.E.V.E.A.L. model (N = 93); Blue: patients identified as low-risk in both models (N = 72). (B) Comparing high-risk patients identified by both models (N = 98) and the other patients (N = 232). Significant difference was found between the two groups (log-rank P = 0.001).
